# Radioactive Iodine-Induced Chronic Myeloid Leukemia in a Patient With Graves' Disease: A Case Report

**DOI:** 10.7759/cureus.35295

**Published:** 2023-02-22

**Authors:** Ahmed K Yasin, Mohammad Abu-Tineh, Awni Alshurafa, Khalid Ahmed, Mohammed Abdulgayoom, Mohammad Afana, Amna Gameil, Mohamed A Yassin

**Affiliations:** 1 Department of Internal Medicine, Hamad Medical Corporation, Doha, QAT; 2 Department of Medical Oncology, Hematology, and Bone Marrow Transplant (BMT) Section, National Center for Cancer Care and Research, Hamad Medical Corporation, Doha, QAT

**Keywords:** hematology, chronic myeloid leukemia, case report, graves' disease, radioactive iodine

## Abstract

Therapy-related leukemia is an increasing concern in hematology. One of these substances that showed to increase the incidence of leukemia is radioactive iodine (RAI). We report here a case of radioactive iodine-induced chronic myeloid leukemia (CML) in a patient with Graves' disease, although most cases in the literature were for thyroid cancer. Also, our patient received a very low dose, which is unique compared to previous case reports in the literature.

## Introduction

Chronic myeloid leukemia (CML) is one of the conditions in the group of myeloproliferative neoplasms (MPN) that happens due to the uncontrolled growth of myeloid cells at different stages of maturation. Patients may present in three phases: chronic phase (CP), accelerated phase (AP), and blast phase (BP) or blast crisis (BC). According to the criteria by the World Health Organization, AP is defined as 10%-19% blasts in peripheral blood or bone marrow, while more than 20% blasts are considered as BC [1].

The symptoms of CML are nonspecific and include fever, fatigue, and weight loss, usually as a result of anemia and splenomegaly. With progression to BC, the symptoms may become more severe and may include bone pain and bleeding. However, half of the patients in CP CML are asymptomatic and may be diagnosed after routine blood tests [1,2].

The diagnosis of CML is made by histopathology, cytogenetics, and the detection of the BCR-ABL1 fusion transcript by reverse transcriptase-polymerase chain reaction (RT-PCR) or of the Philadelphia chromosome translocation (9;22) by fluorescence in situ hybridization (FISH) [3].

Therapy-related leukemia is defined as the occurrence of leukemia following exposure to radiation or hematotoxins. One of these is radioactive iodine (RAI), which was highlighted in multiple case reports as a possible risk for developing CML. And some reports suggested a linear relationship between the dose of RAI and the risk of CML [4]. Here, we report a case of CML following a very low dose of RAI, which shed the light on this subject.

## Case presentation

A 41-year-old Filipina female patient with a known case of coronary artery disease, hypercholesterolemia, and vitamin D deficiency was diagnosed with diffuse hyperthyroidism (Graves' disease) in 2013 by typical symptoms of hyperthyroidism, abnormal thyroid function test, and thyroid scintigraphy that showed symmetrical enlargement of the thyroid gland and diffusely increased radiotracer uptake. Laboratory tests at that time are shown in Table 1.

**Table 1:** Initial laboratory tests when the patient was diagnosed with hyperthyroidism. NA: not applicable

Laboratory parameters	Results	Reference range
White blood cells (WBCs)	6.3×10^3^/µL	4.0-10.0
Hemoglobin (Hb)	14.7 g/dL	12.0-15.0
Platelets (PLTs)	216×10^3^/µL	150-400
Prothrombin time	12.8 seconds	9.7-11.8
Partial thromboplastin time	37.6 seconds	24.6-31.2
International normalized ratio	1.2	NA
Sodium	137 mmol/L	133-146
Potassium	3.7 mmol/L	3.5-5.3
Adjusted calcium	2.18 mmol/L	2.10-2.60
Phosphorus	1.32 mmol/L	0.80-1.50
Magnesium	0.79 mmol/L	0.70-1.00
Creatinine	41 µmol/L	44-80
Urea	2.8 mmol/L	2.5-7.8
Alanine transaminase (ALT)	19 U/L	0-33
Aspartate transaminase (AST)	14 U/L	0-32
Troponin T	12 ng/L	3-10
Thyroid-stimulating hormone (TSH)	0.01 mlU/L	0.45-4.5
Free T3	14.1 pmol/L	3.4-6.0
Free T4	28.7 pmol/L	9.0-20.0

The patient was treated initially with carbimazole and propranolol with regular follow-up with the endocrine team until 2015 where a decision was made to treat the patient with radioactive iodine therapy. A therapeutical dose of iodine-131 capsule approximately 15.32 mCi was given orally on May 24, 2015.

After that, the patient was kept on medical treatment with carbimazole 5 mg per day for two years, and she went into remission, and since 2017, she was not on any thyroid treatment. In 2019, she was found to have subclinical hypothyroidism (thyroid-stimulating hormone {TSH} was 5.8 mlU/L, and free T4 was 14.7 pmol/L) and started on levothyroxine 25 µg daily.

Upon a routine laboratory test for internal medicine clinic follow-up in August 2022, complete blood count (CBC) was done and showed leukocytosis (white blood cells {WBCs}: 29.7×10^3^/µL), and peripheral smear showed neutrophilic leukocytosis with shift to the left and basophilia (9%), normal red cells, and mild thrombocytosis. This picture was suggestive of a myeloproliferative neoplasm (MPN). So, the patient was referred urgently to the hematology clinic.

The patient was reviewed in the hematology clinic in September 2022, she was completely asymptomatic, and her physical examination was unremarkable. Laboratory tests at that time are shown in Table 2.

**Table 2:** Laboratory findings when the patient was reviewed in the hematology clinic. NA: not applicable

Laboratory parameters	Results	Reference range
White blood cells (WBCs)	27.3×10^3^/µL	4.0-10.0
Absolute neutrophil count (ANC)	18.3×10^3^/µL	2.0-7.0
Lymphocyte count	3.0×10^3^/µL	1.0-3.0
Eosinophil count	0.27×10^3^ µL	0.02-0.50
Basophil count	3.28×10^3^/µL	0.02-010
Hemoglobin (Hb)	14.1 g/dL	12.0-15.0
Platelets (PLTs)	643×10^3^/µL	150-400
Prothrombin time	13 seconds	9.7-11.8
Partial thromboplastin time	38.9 seconds	24.6-31.2
International normalized ratio	1.1	NA
Sodium	144 mmol/L	133-146
Potassium	3.7 mmol/L	3.5-5.3
Adjusted calcium	2.27 mmol/L	2.10-2.60
Creatinine	65 µmol/L	44-80
Urea	2.4 mmol/L	2.5-7.8
Total protein	73 g/L	60-80
Albumin	43 g/L	35-50
Alanine transaminase (ALT)	11 U/L	0-33
Aspartate transaminase (AST)	19 U/L	0-32
Alkaline phosphatase (ALP)	56 U/L	35-104
Total bilirubin	5 µmol/L	0-21
Lactate dehydrogenase	442 U/L	135-214
Erythropoietin (EPO)	3.30 mlU/mL	2.59-18.50
Ferritin	51.9 µg/L	12.0-240.0
Folate	11 nmol/L	10.0-70.0
Thyroid-stimulating hormone (TSH)	17 mlU/L	0.30-4.20
Free T4	12.0 pmol/L	11.0-23.3
Vitamin B12	1892.0 pmol/L	145.0-596.0
Hepatitis B surface antigen	Non-reactive	
Hepatitis C antibodies	Non-reactive	
Human immunodeficiency virus (HIV)	Non-reactive	

Further investigations included bone marrow biopsy, which showed findings suggestive of chronic myeloid leukemia (CML) with morphological features of chronic phase. BCR-ABL genetic test was done and showed positive for an e14a2 BCR-ABL1 gene fusion. Fluorescence in situ hybridization was done and showed Philadelphia chromosome translocation (9;22), which is consistent with a diagnosis of chronic myeloid leukemia.

After the confirmation of diagnosis, the patient was started on a tyrosine kinase inhibitor (imatinib 400 mg oral daily) with regular follow-up with the hematology team. Subsequent laboratory tests showed a decrease in WBCs to 8×10^3^/µL, and patient is doing well on treatment and will be followed as per European LeukemiaNet (ELN) 2020 recommendations.

## Discussion

Leukemia in general could be a primary or secondary process to different genetic or environmental factors. Some of the secondary causes are viral infections from Epstein-Barr virus, human T-lymphotropic virus, ionizing radiation exposure, radiation therapy, environmental exposure with benzene, smoking history, history of chemotherapy with alkylating agents, topoisomerase II agents, and others [5].

Radioactive iodine (RAI) was linked to hematologic malignancies in many studies. One large study by Pasqual and her group found that RAI was associated with an increased risk of hematologic malignancies (RR: 1.51; 95% CI: 1.08-2.01), including leukemia (RR: 1.92; 95% CI: 1.04-3.56) [6].

Case reports indicating the occurrence of leukemia after RAI exposure for thyroid cancer patients are increasing, but few case reports described the occurrence of CML specifically after RAI therapy for thyroid cancer [7,8].

In our case report, the patient was treated with RAI for diffuse hyperthyroidism (Graves' disease) not for thyroid cancer, and after reviewing the literature, all case reports of RAI-induced CML were in thyroid cancer patients, so our case looks like a rare presentation.

Some review articles showed that most RAI-induced CML cases were exposed to a dose of more than 100 mCi of RAI [9]. But our case is one of the unique cases in which CML was diagnosed in a patient who received only 15.32 mCi of RAI.

It is still unclear what is the best available therapy for those patients and whether to start with imatinib [10] upfront or dasatinib/nilotinib [11], and it remains one of the unmet needs and unanswered questions regarding CML treatment in different conditions, which we have shed the light on in previous articles such as CML with obesity [12], obesity-related surgeries [13], intermittent fasting [14], hepatitis [15], and tuberculosis [16].

## Conclusions

In conclusion, RAI-induced CML cases are increasing, not only in thyroid cancer but also in Graves' disease cases. Not only large doses but also small doses of RAI can be a risk factor for myeloproliferative complications and should be included in the counselling of patients.
